# Migrasome biogenesis and functions

**DOI:** 10.1111/febs.16183

**Published:** 2021-09-26

**Authors:** Shunbang Yu, Li Yu

**Affiliations:** ^1^ State Key Laboratory of Membrane Biology Beijing Frontier Research Center for Biological Structure School of Life Science Tsinghua University‐Peking University Joint Center for Life Sciences Tsinghua University Beijing China

**Keywords:** cell migration, confocal microscopy, migrasomes, tetraspanin, transmission electron microscopy

## Abstract

The migrasome is a newly discovered organelle produced by migrating cells. As cells migrate, long and thin retraction fibers are left in their wake. On these fibers, we discovered the production of a pomegranate‐like structure, which we named migrasomes. The production of migrasomes is highly correlated with the migration of cells. Currently, it has been demonstrated the migrasomes exhibit three modes of action: release of signaling molecules through rupturing or leaking, carriers of damaged mitochondria, and lateral transfer of mRNA or proteins. In this review, we would like to discuss, in detail, the functions, mechanisms, and potential applications of this newly discovered cell organelle.

## Graphical abstract

Migrasomes are newly found organelles produced by migrating cells and grow on retraction fibers trailing behind the cells. Migrasomes are produced as a result of tetraspanin‐enriched macrodomain accumulation and could be released into the extracellular matrix or engulfed by other cells. Migrasomes could act as carriers of mRNA, protein, or damaged mitochondria, or as chemoattractive sources.

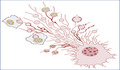

**Abbreviations**
CCCP: carbonyl cyanide 3‐chlorophenylhydrazone
CHO: Chinese hamster ovary cells
CPQ: carboxypeptidase Q
DAOSLIMIT: digital adaptive optics scanning light‐field mutual iterative tomography
DFC(s): dorsal forerunner cell(s)
ECM: extracellular matrix
EOGT: EGF domain‐specific O‐linked N‐acetylglucosamine transferase
FA: focal adhesion
FRAP: fluorescence recovery after photobleaching
MMP: mitochondrial membrane potential
NDST1: bifunctional heparan sulfate N‐deacetylase/N‐sulfotransferase 1
NRK: normal rat kidney cells
PH Domain: pleckstrin homology domain
PIGK: phosphatidylinositol glycan anchor biosynthesis, class K
RF(s): retraction fiber(s)
Tspan: tetraspanin
WGA: wheat‐germ agglutinin

## Introduction

In 2014, we published the discovery of the ‘migrasome’, an organelle produced by migrating cells [1]. Migrasomes form on long tethers called retraction fibers (RFs) that trail behind mobile cells. They undergo a growth period as they receive cytosolic content from the cell body [1, 2, 3]. They are later released into the extracellular environment as the RFs disintegrate [1, 2]. Migrasomes can be acquired by other cells or they may disintegrate and release their contents into the microenvironment [1]. Moreover, cytosolic proteins which do not have a signal peptide can be transported into migrasomes and subsequently released from cells via migrasomes, a process named ‘migracytosis’ [1].

From what has been revealed about migrasomes, it appears that they play important roles in cell–cell communication and maintenance of cellular homeostasis. This review will begin with a summary and discussion of the progress in the field. Then, we will introduce the current methods for studying migrasomes. Finally, we would like to propose our perspectives for future research.

## Discovery and characterization of migrasomes

The discovery of the migrasome was serendipitous. When we were examining transmission electron microscope images of cells in 2012, we noticed pomegranate‐like structures outside cells [1, 4]. These structures were spherical with numerous intraluminal vesicles inside. The diameter of the pomegranate‐like structures was around 2 µm. They were too big to be exosomes and too organized to be the remains of dead cells. Thus, we decided to investigate what these structures were.

**Fig. 1:**
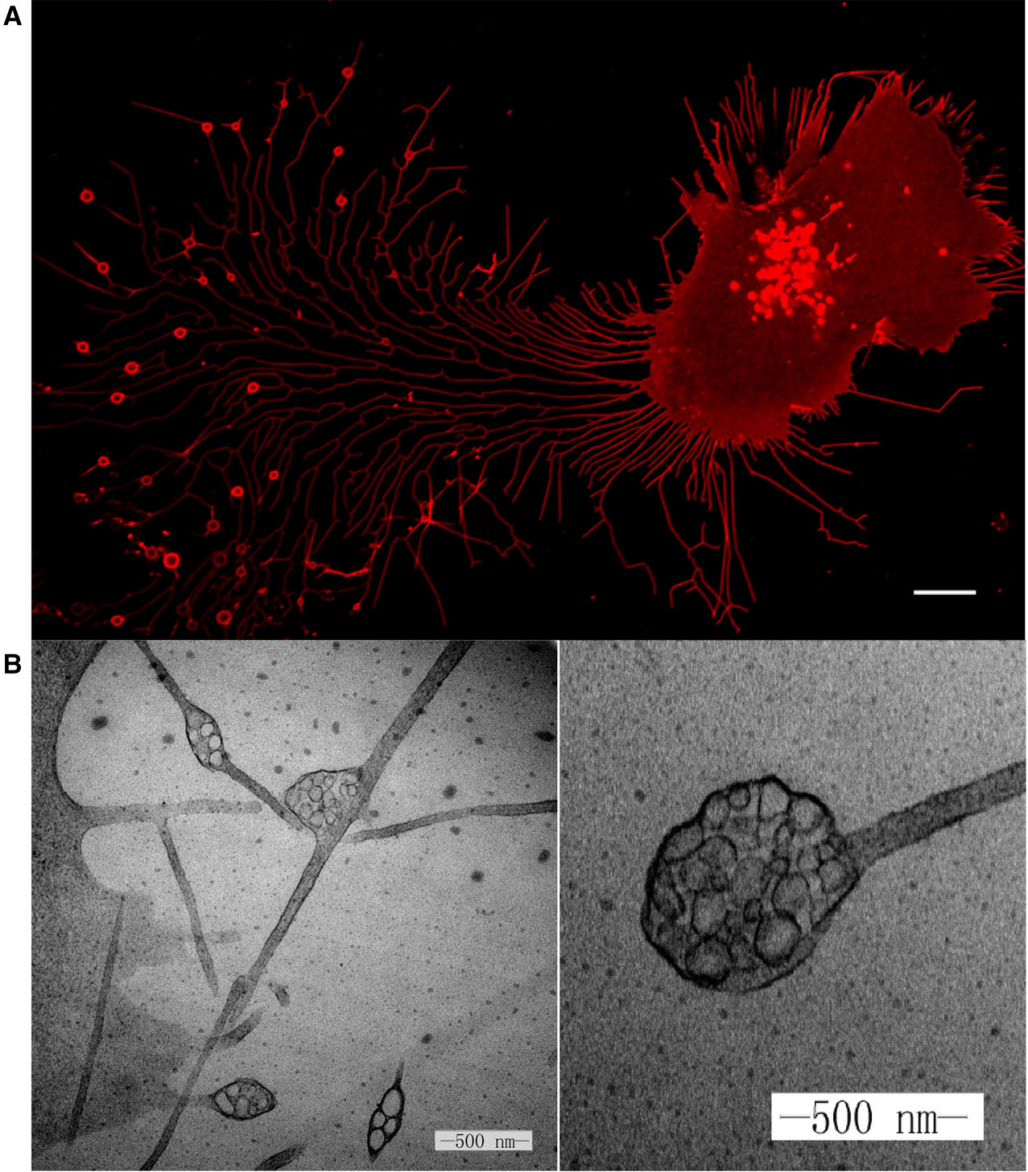
(A) Migrasomes from L929 cells. L929 cells were transfected with Tspan4‐mCherry and visualized by confocal microscopy. Scale bar, 10 mm. (B) Transmission electron microscope image of the pomegranate‐like structures, which we later named migrasomes. Scale bar, 500 nm.

To identify markers for the pomegranate‐like structures, we began by characterizing their contents. Through quantitative mass spectrometry analysis, we identified proteins enriched on migrasomes. The functions of the identified proteins were distributed in areas including cell migration, cell‐substrate adhesion, lipid catabolic processes, protein glycosylation, and glycoprotein metabolic processes. Among the detected proteins, we found that members of the tetraspanin (Tspan) family were highly enriched in migrasomes. Next, we tagged the enriched proteins with GFP and studied their localization. In this way, we identified Tspan4 as a marker for visualizing the pomegranate‐like structures in live cells by confocal microscopy [1]. We observed that as a cell migrates, it leaves long RFs in its wake. Subsequently, large vesicles, with diameters of about 2 µm, form on the tips or branch points of the RFs (Fig. 1A). Through correlative confocal and transmission electron microscopy analysis, we were able to confirm that the vesicles observed in the confocal images were indeed the pomegranate‐like structures observed under the transmission electron microscope (Fig. 1B). In retrospect, we were very lucky to choose Tspan4 as a marker for the pomegranate‐like structures, as we later found that Tspan4 overexpression significantly promotes the formation of these structures, which made the subsequent investigations much easier.

Because the pomegranate‐like structures formed in the wake of migrating cells, we postulated that their formation depended on cell migration. Unsurprisingly, when we inhibited migration, we found that the formation of pomegranate‐like structures was blocked. Following this confirmation that the biogenesis of pomegranate‐like structures was dependent on cell migration, we renamed them as ‘migrasomes’ [1].

## Mechanism of migrasome formation

Migrasomes grow on the retraction fibers trailing behind migrating cells and adhere to the bottom of the culture dish. This means that the production of migrasomes may be regulated by proteins involved in cell–matrix and cell–cell interactions. Indeed, Wu *et al*. found that integrin α5β1 was enriched on the bottom side of the migrasome and could also predict the site of migrasome formation. In cells, integrins are enriched in focal adhesions (FAs) that link the cell to the extracellular matrix (ECM) [5, 6]. However, focal adhesion markers such as paxillin, vinculin, or zyxin do not localize on migrasomes. This indicated that the integrin‐enriched areas on the migrasomes were not focal adhesions, Therefore, Wu and colleagues postulated that the mobilization of migrasomes may depend on specific integrin–ECM interactions. Comparative analysis of the available mass spectrometry data for FAs [7] and migrasomes [8] indicated that FAs and migrasomes share a limited number of enriched proteins—10 proteins in total, which is 1.5% and 1.7% of the total enriched proteins for FAs and migrasomes, respectively. It is worth noting that none of the markers for FAs (paxillin, vinculin, or zyxin) or migrasomes (NDST1, PIGK, CPQ, and EOGT) appeared in the list of shared enriched proteins. Therefore, it is reasonable to conclude that migrasomes are completely different from FAs.

Wu and coworkers found that normal rat kidney (NRK) cells expressing integrin α5‐GFP produced more migrasomes on dishes coated with fibronectin compared to other commonly used coatings. Knockdown of integrin α5 resulted in the impairment of migrasome production. When the investigators overexpressed integrin α3‐GFP, the cells produced more migrasomes on dishes coated with laminin 511 than on dishes with other ECM coatings. Integrin α1‐GFP does not express well in NRK cells, so the researchers used Chinese hamster ovary (CHO) cells instead. Consistent with the previous observations, CHO cells overexpressing integrin α1‐GFP demonstrated enhanced migrasome production on collagen IV‐coated surfaces in comparison with surfaces with other coatings. From these data, Wu *et al*. suggested that the pairing of integrins with their matched ECM ligand determines migrasome formation.

So if integrins mark the site of migrasome formation, how do migrasomes grow? Huang *et al*. (2019) found that tetraspanin family members may play roles far more significant than merely being biomarkers for migrasomes. Tetraspanins are a family of transmembrane proteins which contain 4 transmembrane domains. There are 33 tetraspanins in mammals. Huang *et al*. found that overexpression of 14 tetraspanins can promote migrasome formation. Among them, Tspan4 is highly capable of promoting migrasome formation, and knocking out Tspan4 in MG803 cells reduces migrasome formation. In‐depth investigation of Tspan4‐GFP during migrasome formation revealed that Tspan4 is actively recruited to migrasomes, and the growth of migrasomes is associated with increased intensity of the Tspan4 signal. After migrasomes reach their maximal size, the Tspan4 signal remains steady, which suggests that recruitment of Tspan4 may drive migrasome formation.

**Fig. 2:**
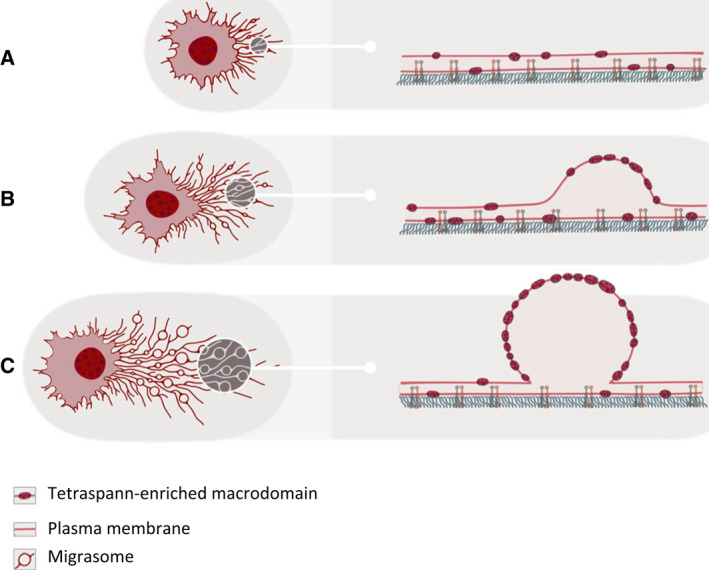
Assembly of tetraspanin‐enriched macrodomains drives migrasome formation. (A) Tetraspanin‐enriched microdomains (dark red ovals, right panels) are evenly distributed along the retraction fiber prior to migrasome biogenesis. (B) When migrasome biogenesis starts, tetraspanin‐enriched microdomains start to assemble on the migrasome formation site. Such assembly leads to formation of a bulge on the retraction fiber. (C) Eventually, the bulge becomes a spherical vesicle when enough tetraspanin‐enriched microdomains are assembled.

On membranes, tetraspanins interact with cholesterol and membrane proteins such as integrins to create tetraspanin‐enriched microdomains [9, 10]. Accordingly, Huang and colleagues found that migrasomes were enriched with cholesterol. Using ultra‐fast resonant scanning imaging and fluorescence recovery after photobleaching (FRAP), they were able to show that during migrasome formation, tetraspanin‐enriched microdomains are assembled into µm‐scaled tetraspanin‐enriched macrodomains. During this assembly process, tetraspanin‐enriched macrodomains bulge out and grow into migrasomes, which suggest that assembly of tetraspanin‐enriched macrodomains may drive migrasome formation (Fig. 2).

To test this hypothesis, Huang *et al*. (2019) set up an *in vitro* system to reconstitute migrasome formation. They deformed giant unilamellar vesicles by mechanical force, generated by liquid flow or by manually pulling the membrane. These forces transformed the giant unilamellar vesicles into long membrane tethers that resembled retraction fibers. If the giant unilamellar vesicles contained Tspan4 and cholesterol, spherical structures resembling migrasomes grew on the membrane tethers. Moreover, Tspan4 and cholesterol were enriched on the migrasome‐like structures. This *in vitro* system provided compelling evidence that assembly of tetraspanin‐enriched macrodomains drives migrasome formation.

How does assembly of tetraspanin‐enriched macrodomains drive formation of spherical structures on a thin membrane tether? To answer this question, the investigators built a theoretical model which they named as the membrane stiffening model. This model predicts that the bulging of migrasomes from retraction fibers is due to the high membrane rigidity of tetraspanin‐enriched macrodomains. The model can be understood through the following analogy: imagine an elastic band, with rigid sections interspersed with soft sections. When the elastic band is stretched, the rigid parts will resist the thinning induced by the stretching force, and thus, they will bulge out relative to the soft sections. Moreover, under certain conditions, many rigid sections can be further assembled into a larger one, thus generating a large spherical structure. The membrane stiffening model predicted that in order to form migrasomal structures, the tetraspanin‐enriched macrodomains needed to have a bending modulus that exceeded that of the adjacent lipid membrane sections by a factor of 5–10. This prediction was confirmed experimentally by measuring the membrane binding rigidity by atomic force microscopy analysis of liposomes containing tetraspanin‐enriched macrodomains.

## Biological functions of migrasomes

**Fig. 3:**
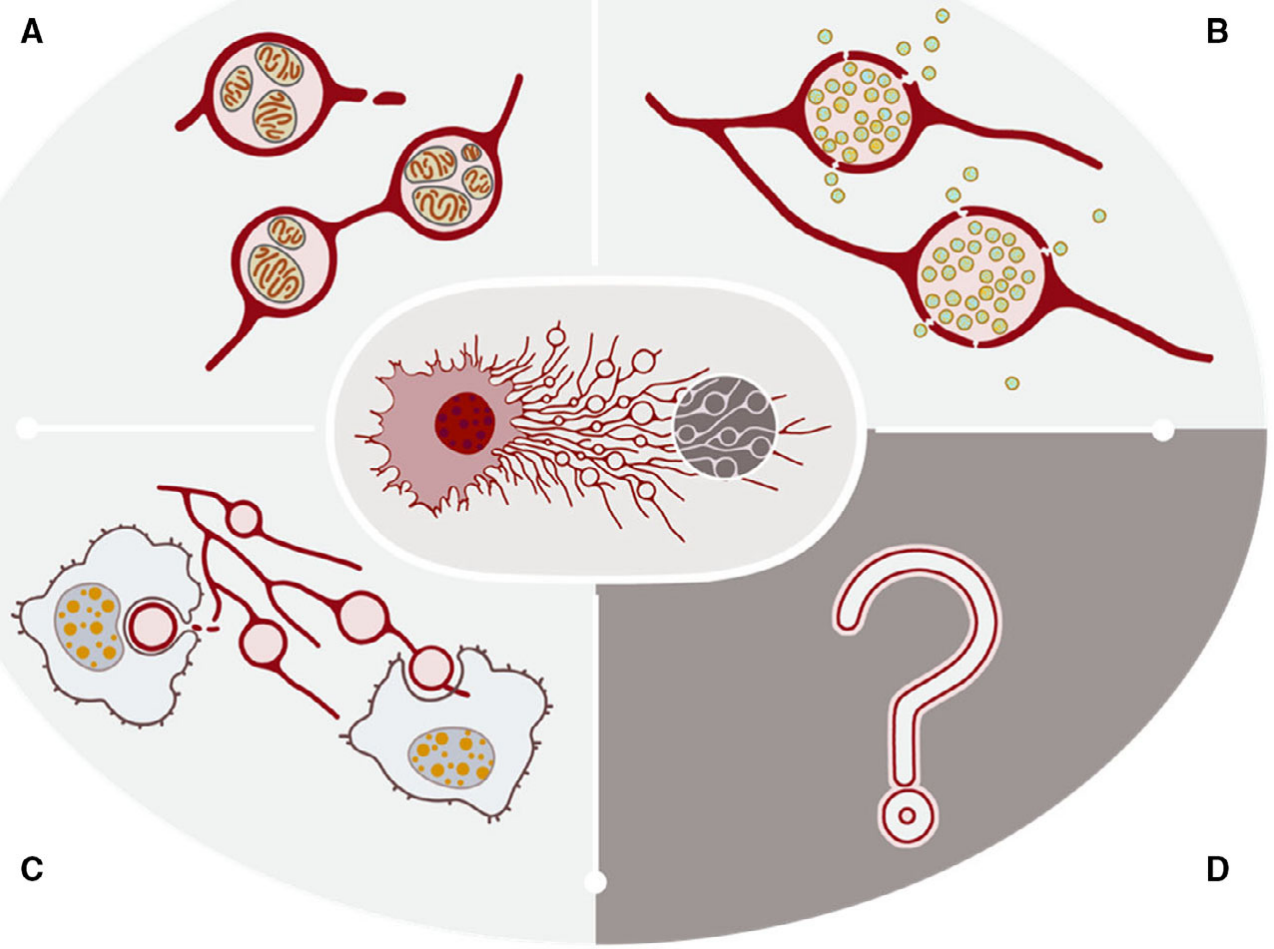
Three modes of migrasome function. (A) Migrasomes dispose of damaged mitochondria; (B) migrasomes act as packets of information with a delivery address; (C) migrasomes mediate lateral transfer of cellular contents. (D) Unknown functions of migrasome still await discovery.

So far, three modes of action have emerged for how migrasomes carry out their biological functions. First, migrasomes act as packets of information which can be delivered to a spatially defined location to signal to the surrounding cells. Secondly, migrasomes act as a garbage disposal mechanism by which damaged organelles are evicted from cells. Finally, migrasomes act to mediate the lateral or horizontal transfer of RNAs and proteins (Fig. 3). In the following paragraphs, we will elaborate on these three modes of action in more detail.

The most important function of migrasomes is probably their ability to serve as packets of information with a delivery address. The paradigm is simple: Migrasomes are enriched with signaling molecules such as chemokines, cytokines, and growth factors. Mature migrasomes can be ruptured or become leaky, thus releasing the signaling molecules, which can then act on the surrounding cells by binding the relevant receptors. This will activate signaling cascades and change the status and behaviors of these cells. Migrasomes can be left in the path of migrating cells, as described above, or they can be delivered to a spatially defined location once formed, as described below. Either way, migrasomes can act as a source of signaling ligands at a specific location long after the cell has migrated away. Thus, migrasomes can integrate spatial and biological information, which is necessary for biological processes involving coordination of action by different migratory cells. Moreover, this ‘latency’ effect of ligand release likely provides another opportunity to fine‐tune ligand‐mediated cell–cell communication. In addition to integrating spatial and biological information, migrasomes are also well equipped to deliver combinatory signals. It is now known that when multiple different signaling pathways are active in a single cell, the biological output is very different from simply summing the biological output of each signaling cascade. This phenomenon is known as combinatory signaling. Migrasomes, by enclosing multiple signaling molecules, can be a perfect carrier for combinatory signals.

The first example of this mode of action came from zebrafish embryonic development. We were able to show that migrasomes played a role in the chemotaxis of dorsal forerunner cells (DFCs) during zebrafish gastrulation [11]. Proper recruitment of DFCs is essential for normal development of left‐right asymmetry. Jiang *et al*. (2019) observed that CXCL12‐enriched migrasomes are generated during gastrulation by mesodermal and endodermal cells. After formation, the detached migrasomes accumulate in a cavity underneath the embryonic shield, thus creating a center for accumulation of CXCL12. Through CXCL12‐mediated chemotaxis, DFCs, which express the CXCL12 receptor CXCR4, are recruited to the embryonic shield. When Jiang and colleagues knocked out Tspan4 or Tspan7, migrasome production was significantly decreased, and the Tspan4‐ and Tspan7‐deficient embryos demonstrated impaired left‐right asymmetry, which is consistent with the known role of DFC recruitment in this process. By injecting migrasomes derived from wild‐type embryos into the impaired embryos, Jiang *et al*. rescued the development of the zebrafish embryos. Thus, migrasomes provide regional cues for embryonic development.

The second mode of action is disposal of unwanted cellular contents. In a recent study, Jiao *et al*. (2021) found that when migratory cells are subjected to mild mitochondrial stress, they can dispose of the damaged mitochondria via migrasomes, a process named as mitocytosis [12]. A striking example of the selectivity of this process comes from experiments using heteroplasmic cells, which carry a population of normal mitochondria and a population of mitochondria with mutant mtDNA. The mutant mtDNA has a large deletion of genes encoding electron transfer chain proteins; thus, mitochondria containing mutant mtDNA are damaged. In migrasomes from these cells, the vast majority of mtDNA inside migrasomes is the mutant form, which suggests that functionally impaired mitochondria are selectively transported into migrasomes. The selectivity appears to come from differential binding of damaged mitochondria to motor proteins. Damaged mitochondria avoid binding to the inward motor protein Dynein, and mitochondrial stressors induce enhanced binding to the outward motor Kinesin 1. Consequently, the damaged mitochondria are transported to the edge of the cell, where they are sent into migrasomes and disposed of. Once they reach the edge of cells, mitochondria are tethered on the plasma membrane via Myosin19, an actin‐based motor protein which is known to bind to cortical actin and mitochondria. The mitochondrial fission protein Drp1 is also required for mitocytosis. Drp1 likely functions in mitocytosis by promoting fission of the damaged mitochondria from the mitochondrial network. Mitocytosis plays an important role in maintaining the mitochondrial membrane potential (MMP) in cells experiencing mild mitochondrial stress; thus, it is a mitochondrial quality control process. The rate of mitocytosis is modest: in L929 cells treated with 2 µm of the uncoupler (CCCP) carbonyl cyanide 3‐chlorophenylhydrazone, mitocytosis evicts 4.4 mitochondria per hour. This is expected, as mitocytosis is triggered by very mild mitochondrial stress. However, consider the self‐amplifying cell death cascade triggered by damaged mitochondria, in which accumulation of even a small number of damaged mitochondria can ultimately lead to cell death. The essential homeostatic role of mitocytosis is supported by evidence from *in vivo* studies using neutrophils as a model. By *in vivo* imaging, Jiao *et al*. found that neutrophils generate large amounts of migrasomes in the circulation. Mitochondria with abnormal morphology are frequently observed in these migrasomes. The researchers then studied Tspan9 knockout mice, in which migrasome formation by neutrophils is reduced. The circulating neutrophils had significantly reduced MMP and decreased viability. These results suggest that disposal of damaged mitochondria is essential for keeping circulating neutrophils alive.

Migration requires energy, which means that migratory cells need more ATP. This in turn necessitates a higher level of mitochondrial respiration. It is known that a higher mitochondrial respiration rate causes higher levels of ROS production and thus greater mitochondrial stress. In this sense, mitocytosis provides an elegant solution. When migratory cells are in the stationary state, they do not move and therefore do not generate migrasomes. At the same time, the cells use less energy, and thus, the mitochondrial activity and the stress load are low, so a balance is achieved. However, when cells start to migrate, migrasomes are generated to mediate mitocytosis, and this balances out the higher mitochondrial stress load cause by the enhanced energy demand. In this way, mitocytosis couples mitochondrial quality control with migration. It is worth emphasizing that mitocytosis seems to be induced only by mild mitochondrial stress. As mentioned above, in L929 cells, mitocytosis is induced by treatment with 2 µm CCCP. When cells are treated with 10 µm CCCP, which is the dose to induce mitophagy, cells do not migrate and do not generate migrasomes. Thus, mitocytosis is not triggered under intense stress conditions. This observation suggests that mitocytosis and mitophagy may possibly serve as a two‐gear system for mitochondrial stress. In this system, mitocytosis deals with mild mitochondrial stress, while mitophagy deals with catastrophic mitochondrial damage. Future study focusing on the relationship between these two mechanisms is required to clarify their roles in mitochondrial quality control.

The third mode of action is lateral or horizontal transfer of cellular contents, including mRNAs and proteins. In *in vitro*‐cultured cells, we frequently observed that migrasomes generated by one cell can be engulfed by surrounding cells. Moreover, cellular contents such as proteins and vesicles can be observed in migrasomes, which suggest that migrasomes can mediate the lateral transfer of materials among cells. Recently, Zhu *et al*. found that migrasomes contain RNAs. Sequencing migrasomal RNAs revealed that the majority of RNAs inside migrasomes are translationally competent full‐length mRNAs. Compared to the RNAs within the parent cell, migrasomes contain a highly enriched subset of mRNAs, including Pten mRNA. When migrasomes containing Pten mRNA were added into Pten‐deficient tumor cell lines, the migrasomal Pten mRNA was translated in the tumor cells, which almost completely abrogated the P‐Akt signal and inhibited the proliferation of these cells. This suggests that lateral transfer of mRNA can modify the recipient cells. One puzzling question remains to be answered: How do endocytosed migrasomes avoid the fate of lysosomal degradation? One possible scenario is that endocytosed migrasomes escape from the endocytic compartment in a manner similar to DNA‐containing liposomes in transfected cells. During transfection, the DNA‐containing liposomes are taken up by endocytosis and enter the endosome compartment. Several hypotheses have been proposed to explain how the DNA is delivered to the nucleus. One popular hypothesis is that components of the liposomes may destabilize the endosomal compartment, thus causing release of the trapped lipid/nucleic acid complexes. It is possible that migrasomes may have a similar effect. Future investigation is needed to address this important question. It is important to note that horizontal transfer by migrasomes has only been observed *in vitro*. Further work is needed to determine whether it can occur *in vivo*, and what its physiological and pathological relevance may be.

## Migrasomes in disease

At this stage, the study of the role of migrasomes in disease is still in its infancy. Up to now, only two groups have reported the possible role of migrasomes in disease. Schmidt‐Pogoda *et al*. (2018) found the existence of migrasomes in the brains of patients suffering from stroke. More recently, Liu *et al*. (2020) found that injured kidney podocytes generate more migrasomes than healthy podocytes. They proposed that urinary podocyte migrasomes could potentially be used as diagnostic markers for detecting early podocyte injury [13]. Additionally, given the known physiological functions of migrasomes in cell–cell communication and in maintaining cellular homeostasis, we expect that in the future migrasomes will be implicated in diseases involving migrating cells such as tumor metastasis, immune disorders, and developmental disorders.

## Methods for studying migrasomes

### Detecting and visualizing migrasomes

Migrasomes were first observed via transmission electron microscopy and scanning electron microscopy [4]. Observation under the transmission electron microscope has remained the most reliable and definitive method for identifying a migrasome. It should be noted that the retraction fibers have an average diameter of 50 nm and are tightly associated with the bottom of the culture dish. However, the diameter of migrasomes can range between 500 and 3000 nm. These parameters must be taken into consideration when studying migrasomes via transmission electron microscopy.

Fluorescence‐based live‐cell imaging by confocal microscopy has been widely applied in migrasome studies. We have shown that Tspan4, integrin, and the pleckstrin homology (PH) domain are reliable markers for visualizing migrasomes [1, 11, 14, 15, 16, 17]. By tagging them with GFP or mCherry, we were able to visualize the migrasome biogenesis process. However, it should be noted that both Tspan4 and integrin, when overexpressed, can enhance migrasome formation. Additionally, not all cells are suitable for transfection.

To allow rapid and convenient visualization of migrasomes, Chen and colleagues searched for dyes that could stain migrasomes. They found that wheat‐germ agglutinin (WGA, a sialic acid‐ and N‐acetyl‐D‐glucosamine‐binding lectin) was an ideal tracker for migrasomes [17]. WGA effectively labels cells, retraction fibers, and migrasomes within a very short time. However, it may be endocytosed by the cells, so it should be kept in the medium, which ensures continuous visualization with an acceptable noise–signal ratio. WGA also works in cell samples fixed with glutaraldehyde (GA) and treated with sodium borohydride (to counter the autofluorescence induced by GA). However, in some samples, zebrafish embryos, for example, WGA does not stain migrasomes well.

In addition to observation by transmission electron microscopy or scanning electron microscopy, we have also identified various protein markers which are enriched in migrasomes—NDST1 (bifunctional heparan sulfate N‐deacetylase/N‐sulfotransferase 1), PIGK (phosphatidylinositol glycan anchor biosynthesis, class K), CPQ (carboxypeptidase Q), and EOGT (EGF domain‐specific O‐linked N‐acetylglucosamine transferase)—in comparison with exosomes [8]. These markers can be analyzed via western blotting to allow rapid determination of the presence of migrasomes (Table 1). It is worth noting that not all these marker must be present to indicate the presence of migrasomes. Depending on the cell type, certain markers may not be detected in migrasome preparations, as the expression levels of these proteins may be very low in the parent cells.

**Table 1:** Markers of migrasome.

Name	Binding specificity/role in migrasome formation
WGA	N‐acetyl‐D‐glucosamine and N‐acetylneuraminic acid residues on plasma membrane glycoproteins
Integrinα5β1	Enriched on the bottom side of migrasomes and anchors migrasomes to the ECM
Tetraspanin Family	Interacts with cholesterol to form tetraspanin‐enriched domains that facilitate the growth of migrasomes
PH Domain	Unknown
NDST1	Unknown
PIGK	Unknown
CPQ	Unknown
EOGT	Unknown

### Isolating migrasomes

Migrasomes are tightly associated with the culture dish, which distinguishes them from all the other extracellular vesicles. Thus, it is crucial to remove the culture medium and wash the cells before the isolation procedure. The isolation procedure is fairly straightforward: Cells and migrasomes are detached by trypsin treatment, cell bodies and large fragments of cell debris are removed by low speed centrifugation, and finally the crude samples are subjected to density‐gradient centrifugation to further enrich the migrasomes. During the isolation procedure, the utmost care must be taken to keep the cells intact; otherwise, the contaminating intracellular organelles will be very hard to remove at a later stage. The detailed practical protocol can be found on our webpage (https://liyu‐lab‐tsinghua.github.io/protocols/).

Please note that all isolation methods result in a highly enriched rather than a ‘pure’ migrasome sample. Thus, careful quality control procedures must be carried out before the samples are used for functional study. These procedures include transmission electron microscope visualization, WGA staining of the migrasomes, and western blotting analysis using markers for migrasomes and markers for potential contaminants, such as organelles and exosomes.

## Future perspectives

Currently, migrasome biology is still in its infancy, so many questions remain to be answered. At this stage, we believe that understanding the mechanism underlying migrasome biogenesis should be the priority. Without a solid understanding of this mechanism, it is going to be very difficult to build a convincing case for functional study. We know that migrasomes are formed by assembly of tetraspanin‐enriched macrodomains; however, we still do not know how the assembly process occurs or how it is regulated. The biogenesis of most organelles is tightly regulated by signaling cascades, which often involve lipid kinases. It remains to be determined whether there is a signaling cascade that governs the formation of migrasomes. Migrasomes contain numerous intraluminal vesicles, but the identity and origin of these vesicles are still unknown. It remains to be determined how these vesicles are formed, sorted, and transported into migrasomes. Beside intraluminal vesicles, migrasomes also contain cytosolic proteins. It is unclear whether these proteins diffuse into migrasomes in a nonselective manner or are selected by a yet‐to‐be‐identified sorting mechanism. Years of effort may be required to answer these fundamental questions.

At present, we know that migrasomes can carry out their functions through three different modes of action. It is very likely that other modes will be discovered in the future. We demonstrated that migrasomes can act as packets of information with a delivery address and that chemokines and cytokines are enriched in migrasomes. It remains unknown whether migrasomes from different cells can enrich different sets of signaling molecules. If this is the case, mapping the enriched signaling molecules for different cell types will help us understand how migrasomes from a particular type of cell may function in a particular biological process. In the second functional mode, migrasomes can be used to maintain cellular homeostasis by evicting damaged mitochondria. At this stage, we do not know whether migrasomes can also be used for disposal of other damaged organelles or unwanted cellular contents. Moreover, the molecular basis for the motor preference of damaged mitochondria is still not known. Finally, for migrasome‐mediated lateral transfer of cellular contents, we need to determine whether this is a physiologically relevant event which can occur *in vivo*.

We found that migrasomes are present *in vivo* in various physiological settings. For example, large numbers of neutrophil‐derived migrasomes are generated in the circulation. Using digital adaptive optics scanning light‐field mutual iterative tomography (DAOSLIMIT), Wu *et al*. (2021) found that the neutrophil‐derived migrasomes demonstrate various behaviors in the circulation: Some can adhere to vessels for a long time, while some are detached quickly after generation [18]. We also observed that migrasomes generated by one neutrophil can be taken up by other neutrophils. At this point, our understanding of the dynamics of migrasomes *in vivo* is still at the descriptive level, and many important questions remain unanswered. For example, we do not know the destination of migrasomes in the circulation. Are they taken up by other cells or do they just break into pieces? What is the half‐life of these circulating migrasomes? What is the determinant for a migrasome to adhere or detach? A systematic investigation of the dynamics of migrasomes *in vivo* is essential for a better understanding of their physiological roles.

So far, the physiological functions of migrasomes have been studied in the context of embryonic development. It is very likely that migrasomes may also play important roles in other biological processes involving migratory cells, including immune responses, angiogenesis, tissue regeneration, and pathological conditions such as tumor metastasis. It will be essential to establish a variety of animal models to study the roles of migrasomes in these biological processes. Finally, recent reports have described the possible role of migrasomes in diseases, and the diagnostic and therapeutic potential of migrasomes have been discussed. Nevertheless, our understanding of migrasomes in diseases is still at a preliminary stage. More solid evidence is needed before the possible diagnostic and therapeutic potential of migrasomes can be considered. Migrasome‐based diagnostic tools and therapeutic approaches can only emerge from a better understanding of the roles of migrasomes in various physiological and pathological processes.

## Conflict of interest

The authors declare no conflict of interest and agree on the submission an publication of this manuscript.

## Author contributions

SY retrieved the literatures, composed the main text, and organized figures and table. LY provided guidance for the primary stage of manuscript writing, provided or drew the figures, proof read the article, provided perspectives, and reorganized or rewrote crucial sections of the manuscript.
